# Ampicillin Treatment of Intracellular *Listeria monocytogenes* Triggers Formation of Persistent, Drug-Resistant L-Form Cells

**DOI:** 10.3389/fcimb.2022.869339

**Published:** 2022-05-12

**Authors:** Virginie Grosboillot, Isabelle Keller, Chantal Ernst, Martin J. Loessner, Markus Schuppler

**Affiliations:** Institute for Food, Nutrition and Health, Swiss Federal Institute of Technology (ETH) Zurich, Zurich, Switzerland

**Keywords:** cell wall-deficient bacteria, L-forms, *Listeria monocytogenes*, intracellular pathogen, antibiotic treatment, antimicrobial resistance, fluorescence *in-situ* hybridization

## Abstract

*Listeria monocytogenes* is an opportunistic intracellular pathogen causing an infection termed listeriosis. Despite the low incidence of listeriosis, the high mortality rate in individuals at risk makes this bacterium one of the most dangerous foodborne pathogens. Reports about a relapse of infection after antibiotic treatment suggest that the bacteria may be able to evade antibiotic treatment and persist as a dormant, antibiotic-tolerant subpopulation. In this study, we observed intracellular generation of antibiotic-resistant L-forms of *Listeria monocytogenes* following Ampicillin treatment of *Listeria monocytogenes* infected cells. Detection and identification of intracellular *Listeria* L-forms was performed by a combination of fluorescence *in-situ* hybridization and confocal laser scanning microscopy. Using micromanipulation, it was possible to isolate single intracellular L-form cells that following transfer into fresh medium gave rise to pure cultures. In conclusion, the results obtained here provide strong evidence that antibiotic treatment of infected host cells can induce the formation of L-forms from intracellular *Listeria monocytogenes*. Furthermore, our results suggest that intracellular L-forms persist inside host cells and that they represent viable bacteria, which are still able to grow and proliferate.

## Introduction

*Listeria monocytogenes* is a rod-shaped, non-spore forming, Gram-positive bacterium. This facultative intracellular pathogen causes listeriosis, a severe disease with a mortality rate of up to 30% in high-risk individuals comprising of young children, elderly people, pregnant women and immunocompromised individuals (Swaminathan and Gerner-Smidt, 2007; WHO, 2018). Serovars 1/2a, 1/2b and 4b are known to cause infections and are responsible for several foodborne outbreaks (Quereda et al., 2021). In the gut, *L. monocytogenes* can cross the intestinal barrier by active invasion of host cells, which is mainly triggered by internalins InlA and InlB. Other virulence factors such as listeriolysin O (LLO) and phospholipases are activated by the low pH inside the phagolysosome and allow the bacteria to escape into the cytoplasm. After the release into the host cell cytoplasm, *L*. *monocytogenes* can multiply and spread to neighbouring cells, driven by ActA-mediated polymerization of host-cell actin (Radoshevich and Cossart, 2018). This strategy enables the pathogen to cross host tissue barriers such as the placental and the blood-brain barrier.

Although the gold standard antibiotic treatment of *Listeria monocytogenes* infections is aminopenicillin or benzylpenicillin alone or in combination with an aminoglycoside (Thønnings et al., 2016), several cases of recurrent listeriosis (within a few weeks and up to several years between the initial infection and the recurring infection) have been reported so far (Ciceri et al., 2017). In some cases, genotyping of the strains causing the initial infection and the recurring infection indicated that they were identical, which was further supported by identical antibiotic resistance patterns. These observations suggest that the strain responsible for the initial infection may have persisted inside the host and caused a recurrent infection to a later time point (Sauders et al., 2001; Rohde et al., 2004; Ciceri et al., 2017). Although molecular confirmation of the *L. monocytogenes* strains causing the initial and the recurrent infection was employed in only a few cases, these findings suggested that the bacteria may not have been completely eradicated by the antibiotic treatment. Instead, they survived the treatment and persisted within infected host cells, thereby escaping detection by standard diagnostic methods. Other studies suggest bone marrow as a reservoir for persistent *Listeria monocytogenes* during and after antibiotic treatment (Hardy et al., 2009), or biofilms forming after surgery and placement of implants (Charlier et al., 2012). Furthermore, *Listeria*-containing vacuoles (Bierne et al., 2018) in which *L. monocytogenes* was shown to enter a slow or non-replicative state were suggested to promote the survival of antibiotic treatment and asymptomatic carriage of the pathogen (Kortebi et al., 2017).

Another possible explanation could be the escape of the bacteria into a cell wall-deficient L-form state. Due to the absence of the cell wall, L-forms are not susceptible to cell-wall active antibiotics such as penicillins and cephalosporins. The morphology of L-form cells appears very heterogeneous because they lack the rigid, shape determining peptidoglycan layer. L-form cells are also highly variable in size and feature an altered metabolic activity (Dell’Era et al., 2009; Leaver et al., 2009; Briers et al., 2012b; Studer et al., 2016a; Studer et al., 2016b). Despite the absence of a mature peptidoglycan structure and the respective division proteins, L-form cells are able to grow and divide through the synthesis of cell membrane lipids that leads to an increase of the cell volume and eventually to the formation of inner and outer progeny vesicles (Dell’Era et al., 2009; Briers et al., 2012a; Mercier et al., 2013; Studer et al., 2016b). However, L-form cell division appears less efficient and much slower compared to walled, parental bacteria, which results in prolonged culture times for L-forms (Dell’Era et al., 2009; Leaver et al., 2009). It was previously shown that L-forms can either be stable, meaning that they are not able to revert back to the walled state, or transient, meaning that upon removal of the inhibitor, they can revert back to a walled, parental form of the bacteria (Kawai et al., 2018; Mickiewicz et al., 2019; Chikada et al., 2021). Both, stable and transient L-forms can be found within the same bacterial culture (Allan et al., 2009).

For decades it was assumed that L-forms may be a possible cause for chronic and idiopathic diseases (Domingue and Woody, 1997). Further support for this hypothesis was provided by the isolation of *Listeria* L-forms from the cerebrospinal fluid of a listeriosis patient (Charache, 1970) or from naturally infected sheep (Prosorovsky et al., 1976). Interestingly, in most cases of relapsing or recurring infections with *Listeria monocytogenes*, Ampicillin or other ß-lactam antibiotics have been used for treatment, often in association with Gentamicin (Levett et al., 1993; Nguyen Van et al., 1994; Lurie et al., 1999; Sauders et al., 2001; Rohde et al., 2004). The same ß-lactam antibiotics are also commonly used to induce L-forms *in vitro*, as they interfere with the cell wall biosynthesis pathway (Dell’Era et al., 2009). Although it was shown that after antibiotic exposure ceases, bacteria may be able to revert back to the walled, pathogenic form, clinical studies were not yet able to provide solid evidence for this hypothesis (Allan et al., 2009).

**Figure 1:**
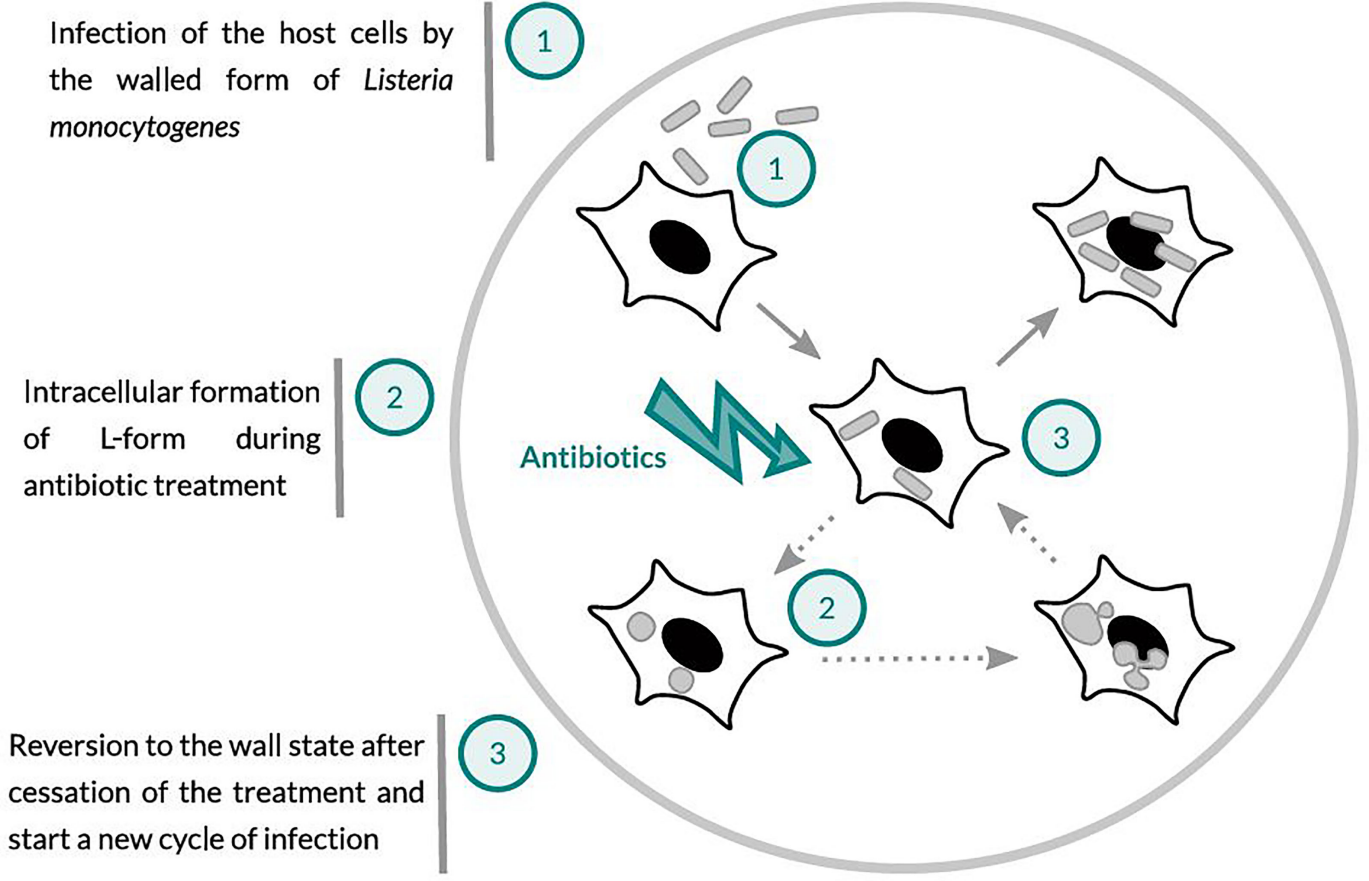
Schematic presentation of the working hypothesis: (1) Host cells infected with *Listeria monocytogenes*. (2) Ampicillin treatment may lead to the conversion of intracellular *Listeria monocytogenes* to wall-deficient L-forms, which are intrinsically drug-resistant and able to survive and persist within the host cells. (3) After the cessation of antibiotic treatment, L-forms may revert back to the walled and fully virulent intracellular pathogen, which can trigger a relapse or recurrent infection.

The aim of this study was to investigate whether treatment of cells infected with *L. monocytogenes* with the ß-lactam antibiotic Ampicillin may induce the intracellular conversion of *L. monocytogenes* to L-forms, which are then able to survive and persist in an intracellular state. In a previous study (Dell’Era et al., 2009) it was demonstrated that peptidoglycan-linked InlA is absent from the surface of *L*. *monocytogenes* L-forms, which prevents invasion and entry in susceptible cell types. This is in accordance with the observation that pathogenicity of *L. monocytogenes* L-forms is completely abolished (Schnell et al., 2014). However, if L-forms are formed intracellularly, they may survive for longer periods of time without producing any signs of infection or pathology. Eventually, after the cessation of antibiotic treatment, the L-forms might revert back to walled, parental *L. monocytogenes*, which regain their full virulence and start a new infection cycle (**Figure 1**).

## Materials and Methods

### Bacterial Strains and Growth Conditions

Strains used in this study are listed in **Table 1**. Parental *Listeria* (wild-type) strains and the walled, revertant L-form variants were grown in liquid Brain-Heart Infusion (BHI) broth (Biolife, Italy) at 37°C with shaking. *Listeria* L-forms were grown at 37°C in liquid DM3 medium, which is an osmoprotective medium originally described by Chang and Cohen (1979), supplemented with 200 μg/ml of Penicillin G (Sigma-Aldrich) when indicated.

**Table 1:** Revertant strains used in this study.

Revertant	Wild-Type	Serovar	Reference
Rev10	*L. monocytogenes* EGDe	1/2a	This study
Rev19	*L. monocytogenes* EGDe	1/2a	This study
Rev30	*L. monocytogenes *WSLC 1042	4b	This study
Rev1	*L. ivanovii *WSLC 3009	5	This study

### Generation of Revertant *Listeria* Strains

The so-called revertant (‘Rev’) strains are variants of *Listeria monocytogenes* that are readily able to switch between the cell wall-deficient L-form and the walled parental state through alteration of culture conditions. To generate Rev strains, 1:10, 1:100 and 1:1000 dilutions of overnight cultures of *Listeria* were inoculated in DM3 soft agar tubes containing 0.8%, 0.5%, or 0.2% agar (Roth, Germany) using a sterile inoculation needle or by dilution of an aliquot of overnight culture directly in liquid DM3. All media were supplemented with 200 μg/ml Penicillin G (Carl Roth, Germany). After several days of incubation, newly formed colonies were successively inoculated into a fresh DM3 culture tube containing lower agar concentrations, until the L-form cells were adapted to grow in liquid DM3 medium. Subsequently, 1:10, 1:100 and 1:1000 dilutions of the L-form cultures were plated on solid DM3 agar medium plates, to support reversion of L-forms to the walled state (Studer et al., 2016a). To verify that liquid cultures did not already contain walled bacteria, the dilutions were also plated on standard BHI agar and checked for the absence of growth. Colonies of revertant bacteria were then isolated from DM3 agar plates, inoculated in glycerol stocks, and stored at -80°C.

### Cell Cultures and Growth Conditions

The placental choriocarcinoma cell line BeWo (ATCC^®^ CCL-98™, passages 195 to 207) was cultured in F-12K Medium (ATCC^®^ 30-2004) supplemented with 10% FBS (Gibco, Thermo Fisher Scientific). Human Brain Microvascular Endothelial Cells (HBMEC), passages 3 to 20, were cultured in RPMI-1640 medium with L-glutamine (Gibco, Thermo Fisher Scientific) supplemented with 1mM Na-Pyruvate (Sigma-Aldrich), 1% MEM non-essential amino acids (Sigma-Aldrich), 1% MEM Vitamin Solution x100 (Sigma-Aldrich), 10% Nu-Serum (Corning^®^, VWR, Switzerland) and 10% FBS (Gibco, Thermo Fisher Scientific), adapted from Silwedel et al. (2018). Both cell lines were cultivated in 75 cm^2^ culture flasks (Bioswisstech, Switzerland) at 37°C and 5% CO_2_ in a humid atmosphere. To maintain the culture monolayer cell cultures were split when 70 to 90% confluence was reached.

### Generation of GFP-Fluorescent Revertant Strains

For each revertant strain, a fluorescent version constitutively expressing GFP was generated by transformation with the site-specific integrative plasmid pPL3e-gfp (Shen and Higgins, 2005).

### L-Form Conversion Rate

Serial 1:10 dilutions of overnight cultures of the revertant strains were plated in parallel onto DM3 agar containing 200 µg/ml Penicillin G and BHI agar media. The DM3 agar plates were incubated at 32°C for up to 3 weeks, and BHI agar plates overnight at 37°C. Colony forming units (CFUs) were determined for both types of plates. The L-form conversion rate was calculated as the percentage of the ratio between the CFU for L-forms and the CFU for walled, parental cells. For this purpose, three biological replicates were performed.

### Cell Line Infection and Intracellular L-Form Conversion

Prior to an experiment, BeWo and HBME cells were grown to 80% confluency. The cells were seeded into 6-well plates (Bioswisstech, Switzerland) for cell lysis, and 2-well Ibidi µ-Slides (Vitaris, Switzerland) or 50 mm Dish (MatTek *In Vitro* Life Science Laboratories, Slovak Republic) for microscopic observation, at a density of 3x10^4^ cells/cm^2^. Invasion assays were performed as previously described (Kühbacher et al., 2014). All media and solutions used were pre-warmed at 37°C. On the day before infection, bacteria from cryo-stocks were inoculated into BHI and grown overnight. Overnight cultures were diluted 10-fold and re-grown until OD_600_ = 0.6-0.8. Approximately 5x10^5^ bacteria were added to the cell lines in serum-free cell culture medium, which corresponds to a multiplicity of infection (MOI) of 10. An aliquot was plated in parallel on BHI agar to determine the number of bacteria (CFU/ml) added to the cell cultures. The culture vessels were incubated for 1 h at 37°C and 5% CO_2_. To stop the infection, the cells were washed with Dulbecco’s Phosphate Buffered Saline (DPBS) (Gibco, Thermo Fisher Scientific) and incubated for 60 min in full medium supplemented with 50 µg/ml of gentamicin to kill extracellular bacteria. For determination of CFU from intracellular bacteria, cells were lysed after 2 h using a solution of 0.5 M Succinate (Carl Roth, Germany) in DPBS supplemented with 0.01% Saponin (Carl Roth, Germany). The lysates were plated on BHI and DM3 agar plates and incubated over night at 37°C. For generation of intracellular L-forms, Ampicillin was added to the cell cultures 7 hours post-infection. Cell culture medium was renewed every two days, and fresh Ampicillin was added every day during the whole period of the experiment. During the initial phase of the cell culture experiments we used 200 µg/ml Ampicillin, because this concentration was used for the generation of revertant *Listeria* strains. Later, it became obvious that the Ampicillin concentration had an impact on the emergence of L-forms *in vitro*, and that concentrations between 25 µg/ml and 50 µg/ml were superior to generate intracellular L-forms.

### Fluorescence *In-Situ* Hybridization

Fluorescence *in-situ* hybridization (FISH) was first tested and established using pure L-form cultures. The bacteria were gently washed three times with a solution of 0.5 M Succinate (Carl Roth, Germany) in DPBS. Fixation was performed with 0.25% Glutaraldehyde (Carl Roth, Germany) for 15 min. After fixation, the sample was washed again three times and incubated for 30 min in 1 mg/ml NaBH_4_ (Carl Roth, Germany) to reduce the autofluorescence caused by the fixative. After another washing step, the samples were dehydrated by incubation for 3 min in increasing concentrations of ethanol: 50%, 80% and 100%, and thus air-dried. *Listeria* genus-specific probes Lis-637 (Schmid et al., 2003) and Lis-1255 (Wang et al., 1992; Wagner et al., 1998) were applied for specific detection and identification of *Listeria* L-forms. Probe EUB338 (Amann et al., 1990; Daims et al., 1999) was used as a positive control, and probe NON338, which is a probe complementary to EUB338 (Wallner et al., 1993) and labeled with the same fluorophore as the *Listeria* probes, was used to indicate the absence of non-specific binding. For hybridization, the four probes (50 ng/µl) were applied individually or in combination in the hybridization buffer (18% NaCl 5 M, 2% Tris/HCl pH 8.0, 30% Formamide, 0.1% SDS 10% [w/v]). Hybridization was performed for 2 h at 46°C. The reaction was stopped by washing for 5 min at 46°C in pre-warmed wash solution (2% NaCl 5M, 2% Tris/HCl pH 8.0, 1% EDTA 0.5M, 0.1% SDS 10% [w/v]). Samples were subsequently washed in distilled water, air-dried, and mounted in Citifluor AF1 (Citifluor; Electron Microscopy Sciences, US) to reduce photobleaching of fluorescent dyes. An almost identical protocol was used for infected cell cultures to detect the possible presence of *Listeria* L-forms inside eukaryotic cells, except that hybridization was performed overnight.

### Confocal Laser Scanning Microscopy

Microscopic inspection of FISH samples was performed using a Leica TCS SPE inverted laser scanning confocal microscope (Leica Microsystems, Germany) equipped with ACS APO 63x/1.30 OIL CS and a HCX PL FLUOTAR 100x/1.30 OIL PH3 objective lenses, unless stated differently. For live imaging, GFP-expressing *Listeria* were excited at 488 nm from a solid-state laser with a power of 1 mW at 100% AOTF (acoustical optical transmission filter). For imaging, the laser intensity was adjusted to 13% AOTF (130 μW), and signals were recorded using a PMT detector with a detection range of 510-550 nm. Microscopy of cells and cultures was performed at 37°C in a temperature-controlled chamber (The Cube & The Box, Life Imaging Services, Switzerland) to maintain optimal conditions for eukaryotic cell lines. For microscopy of FISH samples, the probe EUB338-ATTO488 was excited at 488 nm and the emission signal was collected in a detection range of 510-530 nm. Probes NON338-ATTO532, Lis-637-ATTO532 and Lis-1255-ATTO532 were excited at 532 nm, and the emission signal was collected in a range of 545-560 nm. Hoechst 33258 staining of eukaryotic cell nuclei was excited at 405 nm, and emission recorded in a range of 450-500 nm.

Images shown in **Figure 5** were recorded with a Leica TCS SP8 inverted confocal microscope equipped with a HC PL APO CS2 63x/1.40 OIL objective. Lasers (1 mW at 100% AOTF) were adjusted to an intensity of 13% AOTF (130 μW). The probe EUB338-ATTO488 was excited with the Argon line and the emission signal was collected with a HyD detector in a detection range of 520-525 nm. For probes Non338-ATTO532, Lis-637-ATTO532 and Lis-1255-ATTO532, the signal was collected at 545-560 nm. Transmitted-light microscopy images were obtained using differential interference contrast mode for the 63x objective, and phase-contrast mode for the 100x objective.

Images were acquired using the LAS AF software (Leica Microsystems, Germany). Brightness, contrast, and alpha of images were adjusted using Fiji v2.1.0 (ImageJ software) (Schindelin et al., 2012) to produce the final microscopy images.

### Isolation of Single Intracellular L-Forms Using a Micromanipulator

Isolation and transfer of single GFP-expressing *Listeria* L-forms was performed on a Leica TCS SPE inverted microscope equipped with the TransferMan^®^ NK2 system and a CellTram^®^ Air (Eppendorf, Germany) as previously described by Studer et al. (2016b). In brief, individual fluorescent L-form cells were extracted from a volume of 200 μl L-form culture using an ICSI blunt glass pipette with an inner diameter of 3 µm and a 25° bent extremity (BioMedical Instruments, Germany), and transferred to a fresh culture tube containing antibiotic-free DM3 medium. Cultures were incubated at 32°C up to 3 weeks while regularly monitored for growth of L-forms every week.

The extraction of intracellular *Listeria* L-forms from cell cultures was performed by following the same protocol at 37°C. For this purpose, BeWo and HBME cells were seeded in low, 50 mm Ibidi^®^ µ-Dishes (Vitaris, Switzerland), to provide sufficient room for manipulation with the micro-pipette. Single intracellular L-form cells, or complete eukaryotic cells containing intracellular L-forms were extracted from the cell cultures using an ICSI blunt glass pipette with an inner diameter of 6 or 10 µm. The extracted *Listeria* L-form cells were transferred into fresh, pre-warmed antibiotic-free DM3 broth, and incubated at 37°C.

## Results

### *Listeria monocytogenes* Can Convert Into L-Forms and Revert to the Walled State

In previous studies on the proliferation of cell wall-deficient *Listeria monocytogenes* L-forms (Dell’Era et al., 2009; Studer et al., 2016a; Studer et al., 2016b), stable *Listeria* L-forms have been generated by passaging *L. monocytogenes* for several days to weeks in an osmoprotective medium containing Penicillin G. In order to study the possibility of intracellular conversion of *Listeria* to L-forms in eukaryotic cells, we generated revertant strains that are capable of switching readily back and forth between the L-form state and the parental, walled state. These revertant variants were cultivated and passaged for several weeks on osmoprotective DM3 agar medium containing Penicillin G (Studer et al., 2016a) until it was possible to grow them in liquid DM3.

**Figure 2:**
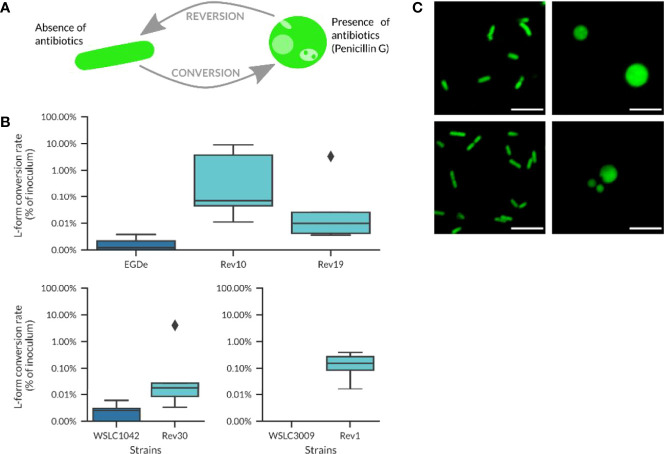
Revertant strains used in this work. **(A)** Schematic overview about the conversion/reversion cycle of *Listeria* revertant strains. Penicillin G was used to trigger the conversion of parental *Listeria* cells to the L-form state. By plating on DM3 agar without drug selection, L-forms revert back to the walled state. **(B)** Conversion rates for different revertant strains compared to their wild-type origin. Revertant strains tend to have a higher conversion rate, although the percentage of converting bacteria is in most cases below 1% and the conversion values are not significantly different between wild-type strains and their respective revertant strains (Welch’s t-test, p-value > 0.05). **(C)** Micrographs of revertant cells at the walled (left) and L-form state (right) following Penicillin G treatment (100 µg/ml). Top row: *L. monocytogenes* strain Rev10 (serovar 1/2a); lower row, strain Rev30 (serovar 4b). Scale bar = 5 µm.

The time needed for conversion to L-forms varied significantly, with an average of 10.5 ± 9.3 days, calculated as the time between first inoculation of the strains on osmoprotective DM3 agar and visible growth of L-forms in liquid cultures. Subsequent culture of L-forms clones on DM3 agar without antibiotics resulted in reversion of L-forms back to walled, parental bacteria, which was confirmed by microscopy. For the revertant strains tested in this study, the reversion process from L-forms back to walled, parental bacteria (i.e., the time between first occurrence of L-forms in liquid medium and the observation of walled, parental bacteria on DM3 agar without antibiotics) was determined as 3.8 ± 1.3 days, which was much faster compared to the time needed for conversion. *Listeria* strains that possessed the ability to convert to L-forms when cultivated in presence of Penicillin G (**Figures 2A, C**, right images) and to revert to their walled state when plated onto DM3 agar plates without antibiotics (**Figures 2A, C** left images) were selected for further analyses. For simplicity, these revertant strains are referred to as Rev strains, whereas the original strains are designated as wild-type strains. The conversion rate could be approximated by plating the strains on osmoprotective medium with Penicillin G. All Rev strains revealed a slightly higher conversion rate than their non-selected wild-type counterparts (**Figure 2B**).

More than 90% of cells from Rev strains showed conversion to L-forms when cultivated overnight in DM3 containing antibiotics, in contrast to only 47% of the respective wild-type strains (n=40). Interestingly, the Rev strains did not show reversion in liquid DM3 without Penicillin G (tested for 3 to 5 passages), while rapid reversion could be observed on solid DM3 agar without antibiotics. This ability to readily switch between the parental and L-form state represented an important property and prerequisite for the investigation of the possibility of intracellular formation of L-forms upon antibiotic treatment.

### Ampicillin Treatment of *Listeria monocytogenes* Infected Cell Cultures Induces Intracellular Formation of L-Forms

**Figure 3:**
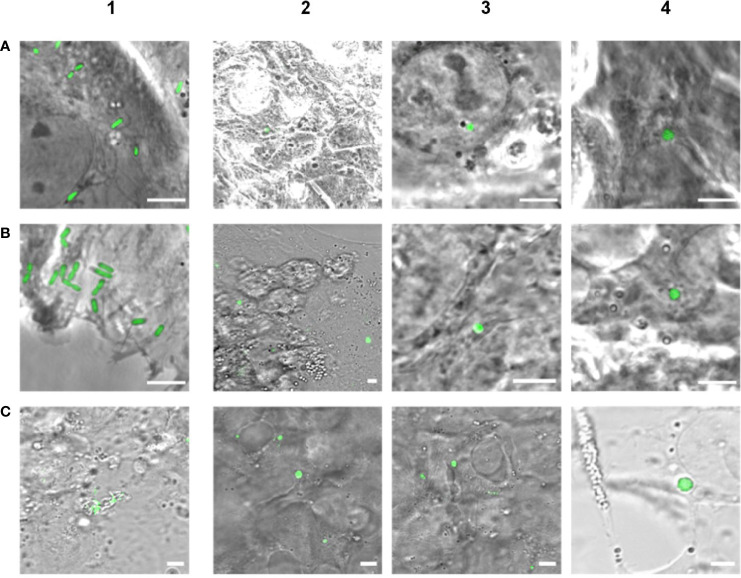
Evidence of intracellular formation of L-forms after Ampicillin treatment (200 µg/ml) of eukaryotic cell lines infected with GFP-labeled *Listeria monocytogenes*. Images in column 1 show infected cells prior to antibiotic treatment, whereas columns 2 - 4 display Ampicillin treated cell cultures. **(A)** Micrographs of HBME cells infected with *L. monocytogenes* EGDe Rev10 before treatment (1), 2 days (2), 4 days (3) and 12 days (4) after Ampicillin treatment. **(B)** Micrographs of BeWo cells infected with *L. monocytogenes* EGDe Rev10 before treatment (1), and 4 days (2-4) after Ampicillin treatment. **(C)** Micrographs of HBME cells infected with *L. monocytogenes* WSLC1042 Rev30 before treatment (1), 6 days (2), and 14 days (3-4) after Ampicillin treatment. Scale bar = 5 µm.

In order to investigate whether intracellular *Listeria monocytogenes* are able to convert to L-forms upon antibiotic treatment and possibly contribute to the persistence of *Listeria monocytogenes* inside host cells as a source for recurring infections, an *in-vitro* cell culture model was established. Human Brain Microvascular Endothelial Cells (HBMECs) were infected with the constitutively GFP-expressing revertant *Listeria monocytogenes* strain Rev10. The presence and fate of intracellular *L. monocytogenes* was assessed by confocal laser scanning microscopy. Seven hours post infection cultures were treated with Ampicillin. Two days later, typical rod-shaped parental *Listeria* were no longer visible in treated cell cultures. However, microscopic inspection of the cell cultures revealed the occurrence of spherical, green-fluorescent structures inside eukaryotic cells between days 2 and 14 after infection. These green-fluorescent, intracellular structures were subsequently identified as *L*. *monocytogenes* (**Figure 3A**). Non-infected and infected cultures without antibiotic treatment served as controls and revealed a lack of intracellular green-fluorescent structures for non-infected cultures, while the cells in infected cell cultures without Ampicillin treatment died off rapidly, most probably due to the high concentration of intracellular *Listeria monocytogenes*.

To validate the observations obtained with HBMECs, the experiment was repeated under identical conditions using a BeWo cell line. Again, Ampicillin treatment, microscopy and plating revealed decreasing numbers of intracellular rods, which completely disappeared about 2 days after treatment. First intracellular L-forms could be observed 2 to 4 days after Ampicillin treatment, which provided more evidence that Ampicillin treatment of cell cultures triggered the intracellular conversion to L-forms (**Figure 3B**). In order to provide additional evidence for the intracellular conversion phenomenon, we also used GFP-expressing revertant strain Rev30 derived from *L. monocytogenes* WSLC 1042 (serovar 4b) for infection of HBMEC cells, followed by an Ampicillin treatment and observation of intracellular L-form generation (**Figure 3C**). In conclusion, our findings provide strong evidence for the emergence of intracellular *Listeria monocytogenes* L-forms upon exposure to the cell-wall active antibiotic Ampicillin, which were able to persist up to 14 days.

### Confirmation of Intracellular *Listeria monocytogenes* L-Forms by Fluorescence *In-Situ* Hybridization

**Figure 4:**
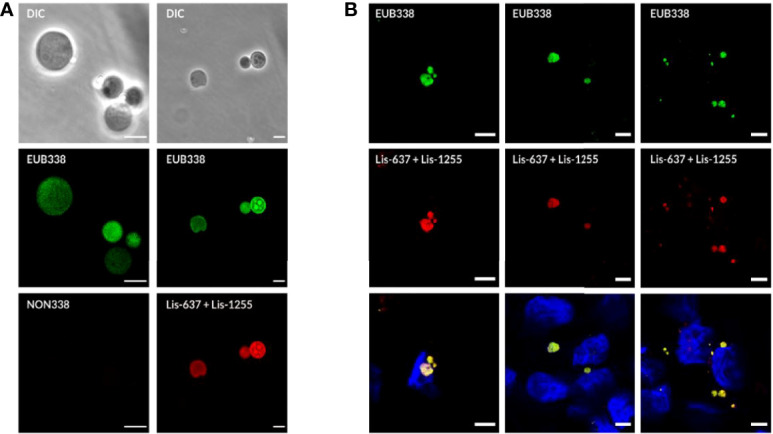
Detection of intracellular L-forms in HBME cells after Ampicillin treatment using fluorescence *in-situ* hybridization (FISH). **(A)** FISH analysis of a pure culture of revertant strain Rev10 L-forms. Hybridization with universal probe EUB338 (green) and *Listeria* genus-specific probes Lis-637 and Lis-1255 (red) resulted in respective fluorescence signals (right column). The absence of a signal after hybridization with probe NON338 (left column), used as a negative control, indicated that the observed signals are not due to background fluorescence or unspecific binding of probes. **(B)** FISH analysis of HBME cells after infection with *Listeria monocytogenes* strains Rev10 (left) and Rev19 (middle), as well as an *L. ivanovii* revertant strain Rev1 (right) and subsequent treatment with Ampicillin (200 µg/ml) for 14 days. Hybridization with universal probe EUB338 (green) and *Listeria* genus-specific probes (red) identified the spherical structures inside HBME cells as *Listeria* L-forms. In the lower row the nucleus of HBME cells is stained in blue and L-forms show a yellow fluorescence due to superimposition of the red signal from the *Listeria* genus-specific probes with the green signal from universal probe EUB338. Scale bar = 5 µm.

Further evidence that the green-fluorescent spherical structures inside infected host cells are true *Listeria* L-forms was provided by a fluorescence *in-situ* hybridization (FISH) approach using fluorescently labelled *Listeria-*specific probes. Because L-forms lack the rigid cell wall providing proper stability during the assay, the standard FISH protocol had to be adapted, and *in-vitro* assays with pure L-form cultures were used to demonstrate the suitability of the adapted protocol for L-form bacteria. The FISH results showed that fixation with Glutaradehyde was suitable and sufficient to preserve the shape of L-form cells. Strong fluorescence signals obtained from *Listeria*-specific probes Lis-637, Lis-1255, and universal probe EUB338, together with the absence of a signal from probe NON338, demonstrated specific labeling of *Listeria* L-form cells (**Figure 4A**).

**Figure 5:**
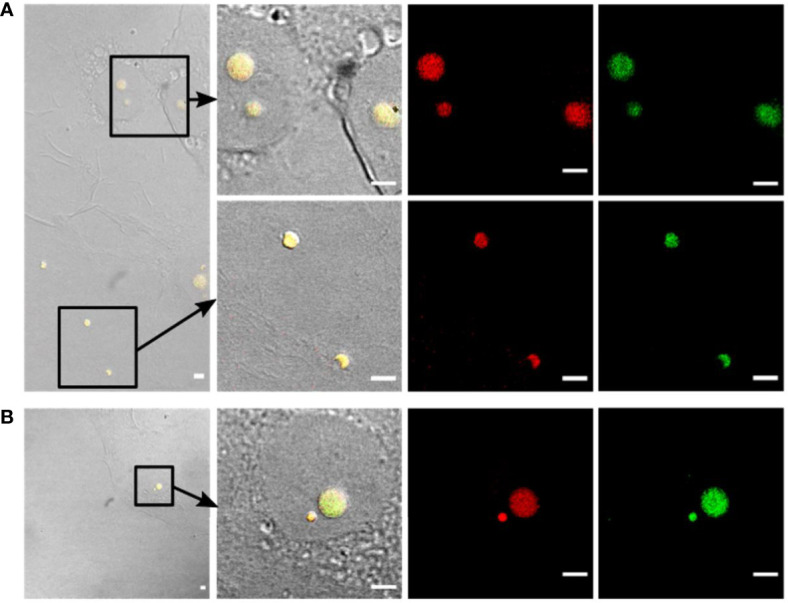
Confirmation of intracellular presence of *Listeria* L-forms in BeWo cells by fluorescence *in-situ* hybridization (FISH). **(A)** BeWo cells infected with *Listeria monocytogenes* revertant strain Rev10 and treated with 25 µg/ml Ampicillin. **(B)** BeWo cells infected with revertant strain Rev10 and treated with 50 µg/ml Ampicillin. Black squares in the DIC images (left) indicate the area of magnification. The first two columns represent a superimposition of DIC images (yellow fluorescence) showing the green GFP-fluorescence and the red signal from the *Listeria* genus-specific probes Lis-637 and Lis-1255. All structures showing green GFP-fluorescence hybridized in parallel with the *Listeria* genus-specific probes (red signals), thus confirming that the observed spherical structures are true *Listeria* L-forms. Scale bar = 10 µm.

In a next step, the FISH protocol was applied for complementary staining of GFP-labelled intracellular L-forms in infected cell cultures following Ampicillin treatment. For this purpose, FISH was performed on Glutaraldehyde fixed cell cultures infected with *L. monocytogenes* Rev10 at different time points after Ampicillin treatment. Hybridization with the *Listeria*-specific probes revealed red-fluorescent, spherical structures inside the host cells which also emitted a green GFP signal. This result provided evidence that the green-fluorescent structures inside the host cells represent true *L. monocytogenes* L-forms (**Figure 4B**, left column). Similar results were obtained with other revertant strains Rev19 and Rev1 (**Figure 4B**, middle and right column), and different eukaryotic cell lines such as BeWo cells (**Figure 5**). In all cases, FISH using the *Listeria*-specific probes resulted in the detection of intracellular L-forms which also showed green fluorescence from GFP (**Figure 5**). In most cases, only one or two L-form cells were observed per microscopic field (**Figure 5B**), which was on average approx. two L-forms per ten eukaryotic cells. Overall, the results from this experiment suggest that the emergence of intracellular *Listeria monocytogenes* L-forms after antibiotic treatment is a common phenomenon.

### Intracellular *Listeria* L-Forms Represent Viable Bacterial Cells

Using the *Listeria*-specific probes for fluorescence *in-situ* hybridization, it was possible to demonstrate that the green-fluorescent structures observed inside the eukaryotic cells are true *Listeria* L-forms. Moreover, the strong FISH signals suggested that these L-forms represent viable cells, because only metabolically active bacteria harbor a sufficient number of ribosomes that contribute to a bright fluorescence signal (DeLong et al., 1989; Kramer and Singleton, 1992; Bouvier and Del Giorgio, 2003).

**Figure 6:**
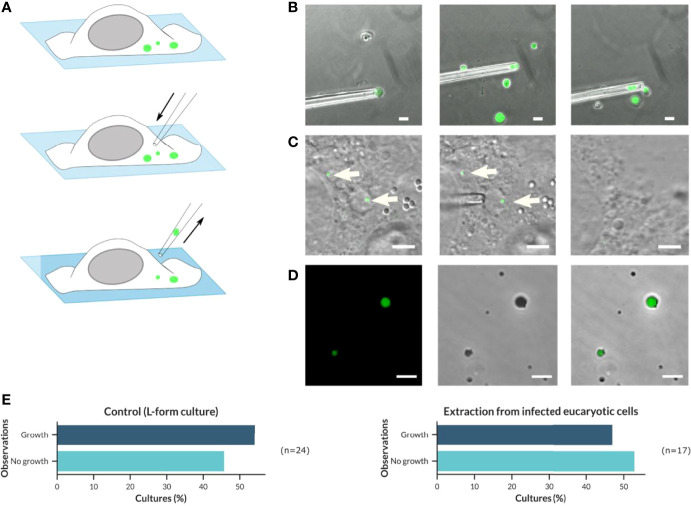
Application of a micromanipulator to isolate and transfer intracellular *Listeria* L-forms from infected host cells to fresh culture medium in order to demonstrate their viability and ability to grow. **(A)** Schematic overview about the procedure of micromanipulator extraction and transfer of intracellular GFP-fluorescent *Listeria monocytogenes* L-forms. **(B)** Proof of concept for extraction and transfer of *L. monocytogenes* Rev10-GFP L-forms from a pure culture to fresh medium using micromanipulation (Scale bar = 10 µm). **(C)** Extraction and transfer of intracellular *L. monocytogenes* Rev10 GFP-expressing L-forms from infected BeWo cells (Scale bar = 5 µm). **(D)** Growth of *L. monocytogenes* Rev10 GFP-expressing L-forms after transfer to fresh culture tubes (DM3 medium without Ampicillin) and incubation for up to three weeks at 32°C was confirmed by confocal laser scanning microscopy (CLSM) (Scale bar = 10 µm). **(E)** Graphical distribution of the proportion of *L. monocytogenes* Rev10 L-forms growing after micromanipulator extraction of L-form cells from pure cultures (left) or BeWo cells (right).

To confirm that the intracellular L-forms represent living cells which are still able to proliferate and grow, a micromanipulation approach was used to extract intracellular L-forms directly from infected host cells, and transfer them to fresh culture medium, which is described in **Figure 6A**. In brief, a blunt glass pipette of 6 to 10 µm was moved towards eukaryotic cells containing intracellular L-forms. Ideally, the cell membrane was punctured by the tip of the glass pipette to allow the L-form cell to be aspired into the pipette. However, it often appeared to be difficult to target and extract a single intracellular L-form cell, due to the movement of the cytoplasmic content when the pressure induced by the glass pipette was applied to the surface of the host cell. Therefore, in many cases, the entire L-form containing host cell was aspired, extracted, and transferred as a whole into DM3 medium without antibiotic (**Figure 6C**).

As a proof of concept, a pure culture of the GFP-expressing *L*. *monocytogenes* EGDe strain Rev10 was grown in DM3 with Penicillin G for one week. From this culture, individual L-form cells were extracted using a micro-manipulator (**Figure 6B**) and subsequently transferred to a fresh tube of osmoprotective DM3 without antibiotics. It turned out that the L-form cells are very sensitive to physical forces and may easily be distorted during intake into the blunt glass pipette. This was particularly true for L-form cells that were larger than the inner diameter of the pipette. However, L-forms that survived the transfer procedure revealed their original shape after careful release into fresh culture medium. Three weeks after transfer, all cultures were evaluated for the presence of green-fluorescent L-forms, which was true for all culture tubes showing already visible turbidity as an indicator for growth (**Figure 6D**). The percentage of cultures showing growth after transfer was defined as the success rate, which was approx. 54% (n=24) of transferred *Listeria* L-forms (**Figure 6E**, left). Due to the absence of antibiotics in the DM3 medium used for this experiment, it can be excluded that L-forms developed from parental bacteria, thus indicating that the L-forms cultures derived from extracted and transferred bacteria.

The same approach was applied for the extraction of intracellular L-forms from BeWo cells infected with GFP-expressing *L. monocytogenes* EGDe strain Rev10 and treated with Ampicillin (**Figure 6C**). The percentage of successful transfers that resulted in cultures showing growth of L-forms was 47% (n=17) (**Figure 6E**, right) and reflected the same range as determined for L-forms isolated from pure cultures. This demonstrated that the intracellular *Listeria* L-forms developed after antibiotic treatment of infected host cells do represent viable bacteria that are still able to grow and multiply, which is a hallmark of the L-form state.

## Discussion

The observation of recurring infections after antibiotic treatment of listeriosis has led to the hypothesis that intracellular *Listeria monocytogenes* escape from antibiotic treatment by conversion to L-forms as a counteracting strategy. However, the conversion of intracellular *Listeria monocytogenes* to L-forms as a consequence of antibiotic treatment and their potential role to serve as persisters could not be demonstrated so far (Onwuamaegbu et al., 2005). Therefore, this study aimed to prove the occurrence of intracellular *Listeria* L-forms after treatment with the antibiotic Ampicillin, as well as to demonstrate the viability of persisting L-forms and their capability to grow and divide as an important prerequisite for their ability to induce relapsing infections.

Domínguez-Cuevas et al. (2012) reported on the requirement for a weakening of the bacterial cell wall in order to enable the escape of the protoplast from the cell wall sacculus as a prerequisite for rod to L-form transition. In this study, we exposed *Listeria monocytogenes* to sub-lethal concentrations of Penicillin G to generate revertant (Rev) strains with the property to readily switch between the L-form and the parental state, generated from two different serovars (1/2a and 4b). The obtained revertant strains demonstrate that the L-form state allows the bacteria to evade and survive antibiotic treatment. Furthermore, it shows that under certain conditions L-forms possess the capability to revert back to the parental state after the removal of the antibiotic. This finding is consistent with previous studies that have shown that different serovars of *Listeria* can be converted to L-forms, which retain the ability to revert back to their walled form (Brem and Eveland, 1967; Brem and Eveland, 1968; Studer et al., 2016a). The availability of revertant strains with the capability to switch between the L-form and the parental state under certain conditions was an important prerequisite for the further investigation of intracellular L-form conversion of *Listeria monocytogenes* in infected cell cultures.

Using cell culture assays and confocal laser scanning microscopy, we could demonstrate that Ampicillin treatment can trigger the intracellular generation of L-forms in host cells infected with GFP-expressing *Listeria monocytogenes*. This important finding confirmed the hypothesis that the use of ß-lactam antibiotics could facilitate the emergence of L-forms inside host cells (Domingue and Woody, 1997) and that these L-forms are able to persist although antibiotic treatment. Because bacterial L-forms may be easily confused with intracellular components of eukaryotic cells, such as vesicles or other spherical structures, the thorough identification of presumptive L-form cells inside the host cells is of utmost importance (Charache, 1970; Mickiewicz et al., 2019). Therefore, a protocol was developed that allowed to prove the identity and viability of the GFP-expressing intracellular *Listeria* L-forms observed by confocal laser scanning microscopy using fluorescence *in-situ* hybridization (FISH) with fluorescent *Listeria* genus-specific oligonucleotide probes. Fluorescence *in-situ* hybridization has been previously applied to identify L-forms isolated from urine samples (Mickiewicz et al., 2019) and showed that the fixation procedure must be adapted accordingly in order to use FISH for specific detection of L-form cells. Using this modified FISH protocol in combination with confocal laser scanning microscopy, it was possible to identify the GFP-expressing spherical structures present in the different cell culture assays as *Listeria* L-forms. Because FISH is based on the hybridization of oligonucleotide probes to the ribosomal RNA, which is a substantial part of the ribosomes, it is broadly accepted that fluorescent signals will only be obtained from cells that have been alive and metabolically active at the time of fixation. In contrast, the fluorescence from GFP is not suitable as a viability marker since the protein remains detectable even in dead cells (Tsien, 1998). However, to provide ultimate proof that the observed intracellular L-forms represent viable cells, additional experiments were performed to extract L-forms from host cells and grow them in culture. Previous studies reported that the extraction and isolation of L-forms from infected human or animal tissue represents a challenging task (Louria et al., 1967; Prosorovsky et al., 1976), which appeared to be more successful for cultures obtained from body fluids (Charache, 1970; Mickiewicz et al., 2019). A possible explanation for the failure of the extraction from tissue samples and cell cultures might be the vulnerable character of the cell wall-deficient L-form cells outside the osmoprotective conditions of their host cells. Sudden changes in the osmolarity or lytic enzymes released during the lysis of host cells may result in the lysis of L-form cells or at least can inhibit their growth, thus having a negative impact on the recovery and culture of L-forms.

In order to overcome the obstacles linked to the extraction of L-forms from cell cultures, a micromanipulator approach was established and applied for target-oriented extraction of intracellular L-form cells and their subsequent transfer into fresh osmoprotective DM3 medium. Using this approach, approx. 50% of the extracted L-forms showed growth after their transfer to fresh DM3 medium. This percentage was even comparable to the percentage obtained for the direct transfer of single L-form cells from a pure culture to fresh medium using a micromanipulator. These results indicate that the low transfer efficiency is not due to the intracellular location of the L-forms and a diminished capability of the L-forms to survive inside the host cells. In contrast, the low transfer efficiency may be explained by the metabolic heterogeneity of L-form populations, as some of the extracted L-forms might not have been metabolically active (Briers et al., 2012b). Another assumption is that a certain number of L-forms in a pure culture may have lost their ability to proliferate as hypothesized in a previous study using micromanipulation for the transfer of L-forms from pure cultures to fresh medium, which reported growth for only 38% of transferred L-forms (Studer et al., 2016a). Furthermore, it might happen that L-forms get lost or were damaged during the transfer procedure, e.g., by sticking to the glass pipette wall or bursting inside the glass pipette. However, the fact that at least half of the extracted L-forms showed growth after transfer to fresh medium demonstrates that the observed intracellular L-forms are truly viable cells that survive antibiotic treatment and persist inside the host cells. This finding is consistent with results from a previous study, which demonstrated that *Listeria* L-forms can persist inside macrophages for several weeks, thus corroborating the hypothesis that bacterial L-forms may play a role as intracellular persisters (Schnell et al., 2014). For *Bacillus subtilis* and *Staphylococcus aureus*, it was also reported that L-forms may occur in macrophages (Kawai et al., 2018) and Green et al. (1974) suggested intracellular reversion of *Streptococcus faecalis* L-forms back in 1974.

The results from this study provide strong evidence for the ability of intracellular *Listeria monocytogenes* to convert to L-forms in non-phagocytic cells after antibiotic treatment and that the pathogen may persist as viable L-forms inside the host cells. However, the intracellular reversion of *Listeria monocytogenes* L-forms to parental bacteria could not be observed, which was probably due to the low frequency of L-forms that developed and persisted inside the host cells and the restriction of the duration of monitoring the fate of intracellular L-forms as a result of the limited viability of host cells in cell cultures. Taken together, the results obtained in this study demonstrate that Ampicillin treatment can trigger the emergence of intracellular *Listeria* L-forms. Thus, these settings can be used for a more in-depth analysis of intracellular *Listeria* L-forms, as it is assumed that their behavior inside host cells is largely different from *in-vitro* culture (Mattman, 2000). Furthermore, it was shown that intracellular L-forms were not only obtained for the two most clinically relevant serotypes (1/2a and 4b) of *Listeria monocytogenes* (Kathariou, 2002), but also for the animal pathogen *Listeria ivanovii* (Vázquez-Boland et al., 2001).

In conclusion, the results from this study demonstrate that the development of intracellular *Listeria monocytogenes* L-forms can be induced by antibiotic treatment of infected cell cultures. Furthermore, L-forms can persist as viable cells that keep their property to grow and divide outside their host cells. Although the conditions under which L-form cells revert to their walled state are yet to be investigated, the current findings on the occurrence of intracellular *Listeria* L-forms support the hypothesis that they are likely to play a role in the persistence and resurgence of listeriosis and chronic infection. Thus, the evidence-based findings from this study suggest the need for an improved antibiotic treatment regime for listeriosis that may take into account the presence of intracellular cell wall-deficient L-forms of *Listeria monocytogenes*.

## Data Availability Statement

The raw data supporting the conclusions of this article will be made available by the authors, without undue reservation.

## Author Contributions

VG, MS, and ML conceived and designed the experiments. VG, CE, and IK performed the experiments. VG, IK, MS, and ML analyzed the data. VG prepared the original draft of the manuscript. MS and ML performed manuscript revision and editing. All authors contributed to the article and have read and approved the submitted version.

## Funding

This work was funded by the Swiss National Science Foundation SNF, project number 170042.

## Conflict of Interest

The authors declare that the research was conducted in the absence of any commercial or financial relationships that could be construed as a potential conflict of interest.

## Publisher’s Note

All claims expressed in this article are solely those of the authors and do not necessarily represent those of their affiliated organizations, or those of the publisher, the editors and the reviewers. Any product that may be evaluated in this article, or claim that may be made by its manufacturer, is not guaranteed or endorsed by the publisher.
